# T-Cell Progenitors As A New Immunotherapy to Bypass Hurdles of Allogeneic Hematopoietic Stem Cell Transplantation

**DOI:** 10.3389/fimmu.2022.956919

**Published:** 2022-07-07

**Authors:** Pierre Gaudeaux, Ranjita Devi Moirangthem, Aurélie Bauquet, Laura Simons, Akshay Joshi, Marina Cavazzana, Olivier Nègre, Shabi Soheili, Isabelle André

**Affiliations:** ^1^ Human Lymphohematopoiesis Laboratory, Imagine Institute, INSERM UMR 1163, Université Paris Cité, Paris, France; ^2^ Smart Immune, Paris, France; ^3^ Department of Medicine V, Hematology, Oncology and Rheumatology, University of Heidelberg, Heidelberg, Germany; ^4^ Department of Biotherapy, Hôpital Universitaire Necker-Enfants Malades, Groupe Hospitalier Paris Centre, Assistance Publique-Hôpitaux de Paris, Paris, France; ^5^ Biotherapy Clinical Investigation Center, Groupe Hospitalier Universitaire Paris Cité, Assistance Publique-Hôpitaux de Paris, INSERM CIC 1416, Paris, France; ^6^ Imagine Institute, Université Paris Cité, Paris, France

**Keywords:** allogeneic hematopoietic stem cell transplantation, T-cells, immune reconstitution, T-cell progenitors, immunotherapy, thymus, immunodeficient, immunocompromised

## Abstract

Allogeneic hematopoietic stem cell transplantation (HSCT) is the treatment of preference for numerous malignant and non-malignant hemopathies. The outcome of this approach is significantly hampered by not only graft-versus-host disease (GvHD), but also infections and relapses that may occur because of persistent T-cell immunodeficiency following transplantation. Reconstitution of a functional T-cell repertoire can take more than 1 year. Thus, the major challenge in the management of allogeneic HSCT relies on the possibility of shortening the window of immune deficiency through the acceleration of T-cell recovery, with diverse, self-tolerant, and naïve T cells resulting from *de novo* thymopoiesis from the donor cells. In this context, adoptive transfer of cell populations that can give rise to mature T cells faster than HSCs while maintaining a safety profile compatible with clinical use is of major interest. In this review, we summarize current advances in the characterization of thymus seeding progenitors, and their *ex vivo* generated counterparts, T-cell progenitors. Transplantation of the latter has been identified as a worthwhile approach to shorten the period of immune deficiency in patients following allogeneic HSCT, and to fulfill the clinical objective of reducing morbimortality due to infections and relapses. We further discuss current opportunities for T-cell progenitor-based therapy manufacturing, including iPSC cell sources and off-the-shelf strategies. These opportunities will be analyzed in the light of results from ongoing clinical studies involving T-cell progenitors.

## Introduction

Allogeneic hematopoietic stem cell transplantation (allo-HSCT) is the only available cure for many hematopoietic malignancies and several non-malignant conditions. The estimated need for allo-HSCT is at least 50,000 patients per year in the EU/US. The successful outcome is highly dependent on the HLA compatibility between donor and recipient with the best clinical results obtained when a matched related donor (MRD) is available. However, the availability of MRDs is limited (approximately 25%–30%); thus, the majority of allo-HSCTs are realized from matched unrelated donors (MUDs) (50%) and mismatched related donors (MMRDs) (20%), whose number is growing since the introduction of post-transplant cyclophosphamide treatment. The outcome of these allo-HSCTs from alternative donors is significantly hampered by complications such as infections, GvHD, and relapses. In fact, as reported by the Center for International Blood and Marrow Transplant Research (CIBMTR) and the European Group for Blood and Marrow Transplantation (EBMT), infections and relapses are the main causes of mortality within the first 100 days post-HSCT (1–3). Despite the advances in supportive care and in different methods for *ex vivo* manipulations of the graft (4, 5), the clinical outcome of allo-HSCT still represents an unmet medical need.

The key issue to be solved is the profound and long-lasting post-transplant immunodeficiency due exclusively to the delayed reconstitution of the T lymphoid compartment, which can take more than 1 year, especially in adult patients. Indeed, competent immune response following allo-HSCT requires T-cell reconstitution with a diverse naïve T-cell repertoire, which results from *de novo* thymopoiesis from donor HSCs. Numerous clinical studies have shown that early T-cell immune reconstitution, and in particular the early acquisition of a CD4^+^ T-cell compartment at day 100 following HSCT, is associated with a lower risk of infection and, in the malignant setting, lower relapse rates and improved event-free and overall survival rates for both pediatric and adult patients (6–9).

T-cell differentiation proceeds through three major steps. First, hematopoietic stem cells differentiate into lymphoid progenitors able to egress the bone marrow (BM) and enter blood circulation. Then, these progenitors migrate to the thymus where they finalize their commitment toward the T-cell lineage differentiation pathway. Finally, thymocytes begin to express their T-cell receptor (TCR) and undergo positive and negative selections. Thymic homing of BM-derived hematopoietic progenitors is thus a prerequisite for continuous T-cell development, as shown with murine models defective in thymic portal endothelial cells (10, 11). In steady state, thymus seeding progenitors (TSPs) migrate into the thymus in small numbers. They interact with thymic endothelial cells, allowing their differentiation into early thymic progenitors (12, 13). With age, both murine and human hematopoietic stem cells are biased towards the production of non-lymphoid cells (14, 15) contributing to a decrease in circulating TSP populations (16). Furthermore, the intrinsic ability of TSPs to differentiate toward T-cell lineage decreases with time (16, 17). Finally, any factor altering the thymic niche results mainly in T-cell-mediated cellular immunity defects, predisposing patients to infections and autoimmune diseases. The thymus is extremely sensitive to various factors and agents, such as stress, infections, or certain medications (18, 19). Thymic impairment has notably been linked to diminished production of chemokines, such as CCL21 and CCL25, involved in the migration of TSPs toward the adult murine thymus (12, 20–22). Aging also induces progressive regression of thymic size and structure, termed thymic involution, resulting in altered thymopoiesis (23). Increased adiposity and fibrosis of the organ and the reduction of cortical volume lead to a disorganized microenvironment associated with reduced thymic output (14, 23, 24).

In the context of HSCT, TSP supply by the bone marrow has been shown to be a limiting factor for T-cell reconstitution (25–27). The occurrence of GvHD correlates with low TSP counts in the blood 3 months post-transplantation (28–30). Additionally, as a drawback of conditioning protocols, chemotherapy profoundly impairs thymus function by causing thymus atrophy, impacting the efficacy of thymopoiesis, and resulting in a decrease in the production of naïve T cells (31, 32).

**Figure 1:**
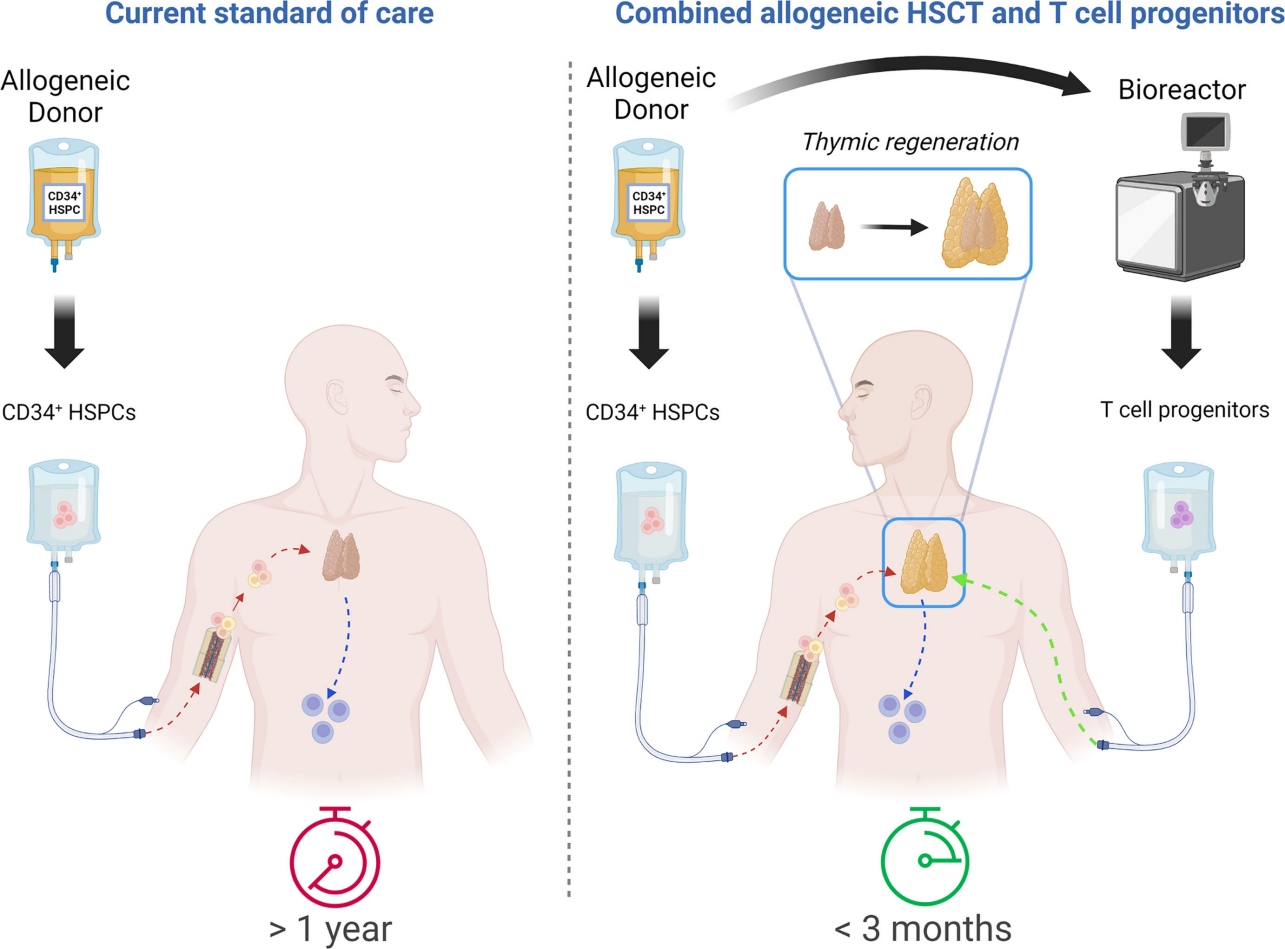
Comparative approach between allo-HSCT as a current standard of care and combination of allo-HSCT with T-cell progenitor transplantation. Timing of fully functional T-cell compartment reconstitution: more than 1 year with current standard of care vs. less than 3 months with combinatorial approach. Created with BioRender.com.

Injection of large quantities of lymphoid progenitors able to directly seed the thymus and generate a wave of thymopoiesis (**Figure 1**) appears as a promising strategy to improve T-cell production after HSCT (33, 34). We will describe the required properties of such lymphoid progenitors, the various types of cells that have been tested in preclinical models, as well as the manufacturing and clinical perspectives.

## Definition of Thymus Seeding Progenitors’ Identity: Blurred Lines Between Physiological Cell Populations

Many circulating hematopoietic progenitors possess T lineage potential, but among them, only a small subset is able to enter the thymus and is therefore considered as true TSP.

In humans, upon reaching the thymus, TSPs upregulate CD7, specifically under the influence of strong intra-thymic Notch signaling induced by the Delta-like Ligand 4 (DLL4)–Notch1 interaction, and differentiate into early T-cell progenitors (ETPs) (35–37). ETPs further differentiate into CD7^+^CD5^+^ thymocytes, which subsequently upregulate CD1a. The CD1a^-^ cells can be subdivided into CD44^+^ and CD44^-^ cells, the latter having full TCRB rearrangements, higher expression of T-cell lineage genes, and solely T-cell developmental potential (38).

Before giving rise to fully mature CD4^+^ or CD8^+^ single-positive T cells (SPs), thymocytes undergo positive selection to select thymocytes with TCR able to bind HLA, and negative selection to eliminate cells that recognize self-antigens. Between the double-negative (DN) and double-positive (DP) stages lies the immature single-positive (ISP) CD4^+^ stage, with no surface expression of CD3 or TCR (39).

The nature of the earliest human TSP is still a subject of debate. Different TSP phenotypes have been proposed previously: a CD34^hi^CD45RA^hi^CD7^+^ phenotype (40), Lin^-^CD34^+^CD10^+^CD24^-^ (16), and cells characterized as Lin^-^CD34^+^CD10^-^CD45RA^+^CD62L^hi^ (41). Two recent studies have performed deeper immune profiling of TSPs using single-cell level analysis. The first one, focusing on the human postnatal thymus, identified two distinct TSPs within the CD34^+^ thymocyte population: CD34^+^CD10^+^CD7^-^ (TSP1), which are Notch-naïve and can represent the most immature progenitors, and CD34^+^CD10^-^CD7^hi^ (TSP2), which are Notch-primed outside the thymus (42). The second study, using human thymus samples, revealed three TSP populations that also have counterparts in the bone marrow: a quiescent HSC-like subset (TSP1), an MPP-like subset (TSP2), and a CLP-like subset with the capacity to commit toward thymocytes much more rapidly than the other two (TSP3) (43).

To colonize the thymus, TSPs must leave the bloodstream through the vascular endothelium, thanks to the combination of multiple mechanisms, most of them having been described in murine models and hypothesized to be conserved in human hematopoiesis. Among them, particular interest has been placed in the adhesion molecule CD44 (44, 45), selectins like P-selectin/PSGL-1 interactions, integrins like α4β1/VCAM-1 and αLβ2/ICAM-1 (46–48), and chemokine receptors CXCR4, CCR9, and CCR7 (22, 49–53). Among other factors that play a role in thymus colonization by TSPs, cytokine receptors such as Flt3 (CD135) and c-kit (CD117) (54) as well as lymphotoxin β-receptor have been documented (10, 55). Progenitors enter the thymus most likely across the cortico-medullary junction area (12).

## *Ex Vivo* Generation of T-Cell Progenitors

Reproducing the pre-thymic process is of high interest given the fact that TSPs are rare and constitute a limiting factor after HSCT. Adoptive transfer of large numbers of *ex vivo*-produced TSP-like cells could accelerate *de novo* T-cell reconstitution post allo-HSCT. This would bypass the initial steps of donor HSC differentiation into TSPs in the bone marrow as well as early induction of the TSPs into T-cell lineage in the thymus. In this case, an *ex vivo* culture system that can provide the essential Notch and cytokine signals to induce HSCs into T-cell progenitor differentiation is a relevant approach to mimic the early T-cell differentiation steps in the bone marrow and the thymus. Moreover, *ex vivo-*generated T-cell progenitors should not, or only incompletely, initiate their TCR rearrangement so that they can undergo thymic selection in the recipient thymus to give rise to self-tolerant T cells.

Initially achieved for fundamental research purposes, fetal thymus organ cultures (FTOCs) depleted of endogenous thymocytes and seeded with human HSCs allowed complete T-cell development, from the DN stage to positive and negative selection (56–58). For less demanding and more standardized culture conditions, feeder cell lines were developed in order to support T-cell differentiation of HSCs (59–61).

The generation of the OP9-DLL1 cell line led to important progress in understanding the different steps of T lymphopoiesis. Both murine and human HSC culture on OP9-DLL1 give rise to the different stages of thymopoiesis, from T-cell progenitor to SP TCRαβ^+^ cells (62–65). Murine and human T-cell progenitors generated using this *ex vivo* co-culture process have both demonstrated their ability to engraft the thymus of NOD-SCID-IL2Rγ^null^ (NSG) mice and further differentiate into T cells (66, 67). Unfortunately, the use of feeder cells, which are most often genetically modified murine cell lines, is not compatible with further clinical T-cell progenitor-based applications. Because the interaction of Notch1 with its ligand is mechanosensitive, immobilization of the ligand on stable surfaces is key to achieving Notch pathway triggering (68). To this end, an IgG Fc fragment-fused DLL1 recombinant protein was generated, enabling, in a feeder-free DLL1-based culture system, efficient expansion of both murine and human hematopoietic progenitors and partly supporting the production of T-cell progenitors (69, 70).

Our team adapted this strategy by developing an hDLL4-Fc recombinant protein compatible with the coating of culture flasks (71). We have further improved this 7-day culture system by a combination of cytokines crucial for stem cell survival and T-cell commitment, allowing the production of a large number of CD34^-^CD7^+^ progenitors. In particular, the addition of TNFα in the culture process resulted in significant improvement in terms of both purity and harvest yield of the final T-cell progenitor product (72). These cells express TCF7, BCL11b, GATA3, and CD3ε; have not yet started their TCR rearrangement; and possess enhanced T-cell potential *in vitro* and *in vivo*, leading to the fast production of mature and polyclonal TCRαβ and γδ T cells upon transplantation in NSG mice (72), similarly to T-cell progenitors produced in 14 days with hDLL4-Fc, IL-7, SCF, and VCAM-1 (73).

Notably, this culture process can be applied to all sources of HSC (such as umbilical cord blood and blood of mobilized donors) (72, 74). It was translated to the clinic and is currently being tested in pediatric and adult patients undergoing HSCT for hemopathies or severe combined immune deficiencies (NCT04707300, NCT03879876, and NCT04959903).

Other teams have compared the effectiveness of DLL4-coated beads with DLL4-coated culture flasks, with the goal of optimizing cell manufacturing (75). However, cell cultures remained static in their study, which is incompatible with actual large-scale bioreactor production. Mechanosensitive Notch1–DLL4 interaction seems not to be sufficiently efficient in a bead–cell complex formed in suspension. Although it does not represent an obstacle for the treatment of patients included in the ongoing clinical trials, this observation underlines the new challenges to be overcome for large-batch production of such T-cell progenitors (e.g., off-the-shelf banks).

## T-Cell Progenitors and Thymic Crosstalk: Boosting Thymopoiesis by Thymus Regeneration

The thymus has a complex structure and encompasses numerous cell types, including thymic epithelial cells (TECs) as well as dendritic cells (DCs), macrophages, fibroblasts, vascular endothelial cells, and connective tissue cells (76). Besides the acceleration of the thymopoietic process, infusion of T-cell progenitors may favor thymus regeneration through crosstalk between the developing thymocytes and the TECs. Different teams demonstrated the direct influence of thymocytes on the formation and maturation of TECs in murine models (77–79). For instance, T-cell progenitors generated *ex vivo* express a high level of RANKL and, thanks to interaction with its receptor, are able to increase transcript levels for TEC-derived chemokines when cultured in FTOC (74, 80). Singh and colleagues reported that injection of T-cell progenitors led to thymic engraftment and regeneration not only in young murine recipients but also in aged recipients (80).

Mechanisms underlying thymic regeneration and cell populations contributing to it were recently detailed by Cosway and colleagues, highlighting the cooperative work of eosinophils, iNKT cells, and ILC2 in the murine thymus (81). The ILC2 population was linked to the production of IL5 in the thymic microenvironment, resulting in the acceleration of thymocyte replenishment in the organ. Interestingly, Hernández and colleagues recently demonstrated the spontaneous occurrence of ILC2 and T-cell progenitors in the OP9-DLL1 culture system, indicating the apparent ontogenic proximity of these two cell populations (82). This characterization work paves the way for the development of both adaptive and innate cell-based therapies that may be able to synergize in promoting thymic regeneration.

## The Future of T-Cell Progenitors: Scaling Up Production for Broad Clinical Application

One approach for widening the use of T-cell progenitor-based acceleration and enhancement of T-cell reconstitution post-allogeneic HSCT is the availability of off-the-shelf T-cell progenitor sources. Some of the obstacles for this strategy include the unavailability of major histocompatibility complex (MHC)-matched donors (if not autologous), and the time and cost required to generate personalized T-cell progenitor-based immunotherapy. In this context, Zakrzewski et al. have demonstrated that the adoptive transfer of *ex vivo*-generated T-cell precursors in an irradiated recipient with a complete MHC mismatch led to efficient thymic regeneration and functional TCR selection with host-tolerant T cells, while avoiding GvHD (83). These data paved the way for the development of an off-the shelf T-cell progenitor strategy as a universal immunotherapy. Cryopreserved cord blood unit (CBU) cells provide the ideal platform for the generation of these cellular therapy products due to its immediate availability, high proliferation yield during differentiation (72), and the possibility of covering the majority of the population using a limited number of units. By creating an off-the-shelf T-cell progenitor bank from umbilical cord blood CD34^+^ cells, the therapeutic cell product would become immediately available to patients in need, independently of the donor, even when allogeneic HSCT is not possible (e.g., following lymphodepleting chemotherapy, in aging patients as protection against viral infections, or in the context of immunosuppressive therapy).

Induced pluripotent stem cells (iPSCs) could also be a relevant source to produce such off-the-shelf banking: iPSCs can unlimitedly self-renew and possess the potential to differentiate into all cell types, including immune cells (84–86). They can be easily genetically engineered and clonally selected to ensure a homogeneous cell derivative (87). If genetic modifications and clonal selections are needed, master iPSC lines with the desired modifications would be banked as a renewable T-cell progenitor source. T-cell production from iPSCs consists of a series of multiple differentiation steps that includes induction of iPSCs into mesodermal progenitors, mesodermal progenitors into hemogenic endothelial cells (HECs), HECs into CD34^+^ hematopoietic stem and progenitor cells (HSPCs), and HSPCs into T cells in the presence of DLL1- or DLL4-induced Notch signaling (88–90). Multiple studies had successfully reprogrammed somatic T cells into iPSCs and differentiated them back into highly proliferative T cells, while maintaining their rearranged TCRs (91–95). Recently, Flippe et al. developed a feeder-free method to generate HSPCs from iPSCs, which were closely similar to cord blood-isolated CD34^+^ HSPCs and could give rise to T-cell progenitors upon co-culture with OP9-hDLL1/DLL4 murine stromal cells (96, 97). An artificial thymic organoid-based culture system has also been described to generate conventional T cells from iPSCs (98). All these methods are based on stromal feeder cells of murine origin (hDLL1- or hDLL4-expressing MS5 or OP9), which limits their use in the clinic. In an effort to develop clinically compatible methods, feeder cell-free immobilized DLL1 or DLL4 microbead-based techniques have been developed to generate T-cell progenitors and T cells from iPSCs (75, 90). Progenitor T-cell differentiation has also been shown to be improved by the induction of endothelial-to-hematopoietic transition on DLL4 and VCAM1 (99).

However, safety concerns associated with iPSC-based cell therapy should be considered. Risk factors of using iPSC-derived cell products include genetic and epigenetic abnormalities occurring during reprogramming or subsequent culture for maintenance (100–103), their potential to become tumorigenic and immunogenic (104–107), as well as variability across iPSC lines and batches (108).

## Concluding Remarks

After allogeneic HSCT, 18 to 24 months are usually necessary to reconstitute a polyclonal T-cell repertoire. The possibility of using T-cell progenitors to accelerate post-transplant lymphoid reconstitution appeared concomitantly with the identification of thymus seeding progenitors (TSPs) and the development of T-cell differentiation protocols based on Notch, DLL1, and DLL4 ligands. Although TSPs cannot be used as such because of their rarity and the difficulty in isolating them, T-cell progenitors generated *ex vivo* have largely demonstrated their effectiveness, both through congenic studies in mice and through xenogeneic experiments with human cells. More importantly, they also appear to be effective when transplanted into aged mice, which raises the prospect of their use in elderly patients with highly involuted thymuses (83). While this review is written in the context of allo-HSCT, it may be of interest to also highlight perspectives of T-cell progenitor use concomitantly with autologous HSCT: recent findings show that even in autologous settings, immune reconstitution and tolerance is impacted, resulting in the phenomenon of auto-GVHD (109).

Other studies suggest that T-cell progenitors could participate in the regeneration of thymic architecture through cytokines expressed at the progenitor stage and at the CD4^+^ SP stage (110). If this hypothesis is confirmed, the injection of T-cell progenitors could improve thymic function, which would be particularly useful in patients whose thymus has been altered by chemotherapy, irradiation, infections, GvHD, or inflammation, as well as in elderly patients whose thymuses have undergone thymic involution. Moreover, increased recovery of other thymus-dependent lineages such as γδ T cells, NKT cells, or MAIT cells (111–113) could be speculated on the basis of preclinical results (72). These properties of T-cell progenitors are under investigation in ongoing clinical trials in adults treated for malignant hemopathies.

We have developed a GMP-compliant feeder cell-free process, compatible with different HSC sources and allowing the manufacturing of cryopreserved drug products for clinical trials. Combined with the possibility of using T-cell progenitors that are partially incompatible with the recipient, this opens prospects for off-the-shelf biobanks of allogenic T-cell progenitors with centralized production. iPSCs also appear as a source of stem cells for T-cell progenitor manufacturing, but further developments are required before considering clinical applications. The possibility of genetically modifying these cells during production, for example, arming them with a chimeric antigen receptor (CAR), further increases their potential in immuno-oncology.

## Author Contributions

All authors listed have made a substantial, direct, and intellectual contribution to the work, and approved it for publication.

## Funding

This work was supported by the French Institut National de la Santé et de la Recherche Médicale (INSERM), the European Union Seventh Framework Program under grant agreement No. 269037 and No. 261387, the European Union’s Horizon 2020 research and innovation program under grant agreement No. 666908 and No. 811171, and state funding from the Agence Nationale de la Recherche under the “Investissement d’avenir” program (ANR-10-IAHU01), the Paris Île-de-France Region under the “DIM Thérapie génique” initiative, and French Ministry of Health under the 2015 hospital clinical research program. Assistance Publique-Hôpitaux de Paris is sponsoring two clinical trials (NCT04707300 and NCT03879876); Smart Immune is sponsoring the NCT04959903 clinical trial. The Human Lymphohematopoiesis Laboratory and Smart Immune have contracted an Industrial Agreement of Training through Research (CIFRE #2019/0690) granted by French National Association for Research and Technology (ANRT).

## Conflict of Interest

MC, IA, RM, LS, and SS have submitted two patents describing the method of generating T-cell progenitors. MC and IA are co-founders and hold equity in Smart Immune. PG, AB, LS, MC, ON, and SS work at Smart Immune. IA is consulting for Smart Immune.

The remaining authors declare that the research was conducted in the absence of any commercial or financial relationships that could be construed as a potential conflict of interest.

## Publisher’s Note

All claims expressed in this article are solely those of the authors and do not necessarily represent those of their affiliated organizations, or those of the publisher, the editors and the reviewers. Any product that may be evaluated in this article, or claim that may be made by its manufacturer, is not guaranteed or endorsed by the publisher.
